# Thermoelectricity and Thermodiffusion in Magnetic Nanofluids: Entropic Analysis

**DOI:** 10.3390/e20060405

**Published:** 2018-05-24

**Authors:** Thomas J. Salez, Sawako Nakamae, Régine Perzynski, Guillaume Mériguet, Andrejs Cebers, Michel Roger

**Affiliations:** 1 Service de Physique de l’État Condensé, CEA, CNRS, Université Paris-Saclay, 91191 Gif sur Yvette CEDEX, France; 2 Laboratoire Physicochimie des Electrolytes et Nanosystèmes Interfaciaux (PHENIX), Sorbonne Université, CNRS, 4 Place Jussieu, F-75005 Paris, France; 3 MMML Lab, Faculty of Physics and Mathematics, University of Latvia, Zellu-8, LV-1002 Riga, Latvia

**Keywords:** thermoelectricity, thermodiffusion, nanofluids, colloids, seebeck coefficient, thermogalvanic cells

## Abstract

An analytical model describing the thermoelectric potential production in magnetic nanofluids (dispersions of magnetic and charged colloidal particles in liquid media) is presented. The two major entropy sources, the thermogalvanic and thermodiffusion processes are considered. The thermodiffusion term is described in terms of three physical parameters; the diffusion coefficient, the Eastman entropy of transfer and the electrophoretic charge number of colloidal particles, which all depend on the particle concentration and the applied magnetic field strength and direction. The results are combined with well-known formulation of thermoelectric potential in thermogalvanic cells and compared to the recent observation of Seebeck coefficient enhancement/diminution in magnetic nanofluids in polar media.

## 1. Introduction

The existence of thermoelectric effects in liquid electrolytes is long known [1]. However, their use as a potential source of renewable energy was considered unlikely, due to their low ionic conductivity compared to the solid semiconductor counterparts. Such a view is quickly changing since the discovery of high thermogalvanic Seebeck We note that strictly speaking, the term “Seebeck effect” describes a thermoelectric energy conversion phenomenon observed in the solid-state materials. However, the term is now commonly extended to refer to the “temperature coefficient” [1] in thermogalvanic (temperature dependent redox reaction) cells containing liquids [2]. To be in-line with this trend, here we loosely employ the term "Seebeck" to delineate the thermoelectric potential generation across two electrodes in thermogalvanic cells, due to both the oxidoreduction reactions and the internal thermoelectric field (see next section for more precision)) effect (in the order of 1 to 10 mV/K) in complex liquids. (See for example: ionic liquids ([3,4,5,6,7]), aqueous ([8,9,10,11]) and mixed-electrolytes ([12,13]), and gelled electrolytes ([14,15])).

More recently, an enhanced Seebeck coefficient was reported in another type of complex fluid; namely, charged colloidal solution known as ionic nanofluids (but not ionic liquids) [16], which is believed to be related to the thermodiffusion (Soret effect) of dispersed colloidal particles. Indeed, thermodiffusive behavior (Soret coefficient) of charged colloidal particles are know to depend strongly on the size, the shape, the surface charge of charged colloidal particles as well as on the surrounding ionic environment and the resulting internal thermoelectric field (for example, see [17,18,19,20,21]).

In solid materials, thermoelectric potential can be calculated from the out-of-equilibrium thermodynamics where the energy and mass flux are expressed in terms of Onsager coefficients $L_{ij}$ (see for example, [22]). Unlike in solids where there is usually only one type of charge carriers (electrons or holes), there are at least two if not more charge carriers (ions and colloidal particles) in any given liquid electrolyte. These carriers are all subject to thermodiffusion and interact among themselves, complicating considerably the theoretical analysis of such systems [23,24]. In most thermogalvanic cells, the Seebeck potential due to the thermodiffusion of small ions is much smaller (only a few percent) than that of the thermogalvanic term and thus ignored (see, for example, [2,25]). In ionic nanofluids containing large (nanometric) heat and charge carriers, however, such simplification is no longer valid.

The goal of the present article is therefore to provide analytical model linking the well-known macroscopic phenomena in liquid electrolytes; i.e., Soret and Seebeck effects, to the physical parameters (charges, diffusion coefficients, etc.). Our particular attention is given to magnetic ionic nanofluids where both positive and negative effects were observed depending on the nature of the magnetic particles and of the base fluids.

In the following sections, we will first define a typical thermocell containing ionic nanofluids and its operation condition being considered. Two distinct sources of Seebeck potential are then described; which are, thermogalvanic Seebeck effect and internal Seebeck effect due to the thermodiffusion of charged species. The magnitude of the latter depends strongly on three key parameters; the Eastman entropy of transfer ($\hat{S}$), the electrophoretic charge number (ξ) and the diffusion coefficient *D* whose values we aim to identify through subsequent entropic analysis.

## 2. Thermogalvanic Cell

**Figure 1:**
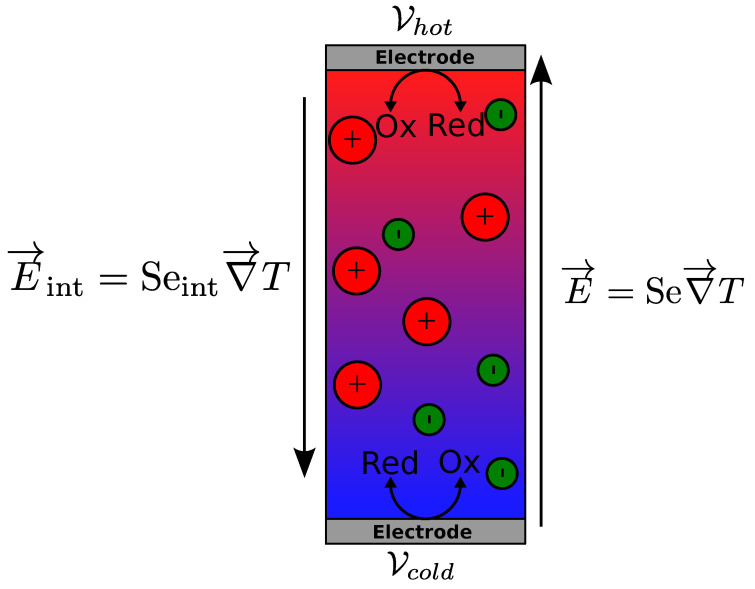
Schematic representation of two electric fields in a thermocell. The internal electric field ${\vec{E}}_{\mathrm{int}}$ is created in the solution volume by thermodiffusion of ions. The thermogalvanic electric field $\vec{E}$ is created between the two electrodes. The hot and cold electrode potentials are $V_{\mathrm{hot}}$ and $V_{\mathrm{cold}}$ : $\left|\vec{E}|=|\frac{V_{\mathrm{hot}}-V_{\mathrm{cold}}}{l}\right|$, *l* being the distance between the electrodes.Electric field in a thermocell

A simple thermogalvanic cell considered here is filled with a solution composed of a liquid (not an ionic liquid, and considered as a continuous medium), charged (magnetic) particles with surface charge number of ξ, counterions (for electric neutrality of the solution) and the redox couple molecules. The two ends of the cell are sealed hermetically with identical and metallic electrodes. The cell is assumed to be heated from the top so that no convection occurs. An electrical potential, ΔV, appears across these electrodes upon application of a temperature gradient, Δ*T* (see Figure 1) due to both the thermogalvanic effect of redox reactions and the internal electric field induced by the charged species in the bulk. The total Seebeck coefficient is defined by:

(1) $$\mathrm{Se}=-\frac{\Delta V}{\Delta T}$$

with V being the potential at each electrode. (The Seebeck coefficient is defined here and in the rest of this article as $\vec{E}=\mathrm{Se}\vec{\nabla }T$, hence the minus sign in Equation (1). This definition is often used for theoretical works within the solid-state thermoelectric community [22,26] and has been retained here for an easier comparison with other theoretical works. However, in the thermogalvanic cell community, it is not uncommon to see the following definition: $\mathrm{Se}=\frac{\Delta V}{\Delta T}$).

As stated earlier, in most liquid electrolytes containing only small ions, the thermogalvanic Seebeck coefficient is much larger than the internal one and thus the latter is often ignored. In ionic nanofluids, and in magnetic nanofluids in particular, the internal term (${\mathrm{Se}}_{\mathrm{int}}$) has been shown to make non-negligible contributions to the total Seebeck coefficient (Se) of the liquid.

### Thermogalvanic Seebeck Coefficient

The redox half reaction occurring at a metallic electrode and involving a single electron transfer can be described by:

(2) $$\overset{\mathrm{in}\;\mathrm{solution}}{\overset{︷}{\sum_{j}\nu_{j}A_{j}}}+\overset{\mathrm{inside}\;\mathrm{the}\;\mathrm{conducting}\;\mathrm{electrode}}{\overset{︷}{e^{-}}}=0$$

where $A_{j}$ are chemical species participating in the redox reaction, and $\nu_{j}$ the corresponding stoichiometric numbers. Here we suppose that both reducing and oxidizing species are charged, and no other species (counterions and charged particles) take part in the redox reaction ($\nu_{k}=0$). In a simplest case, i.e., $\nu_{\mathrm{Ox}}=1$ and $\nu_{\mathrm{Red}}=-1$, the reaction is written as:

(3) $${\mathrm{Ox}}^{n-}+e^{-}={\mathrm{Red}}^{(n+1)-}$$

(4) $${\mathrm{Ox}}^{n-}+e^{-}-{\mathrm{Red}}^{(n+1)-}=0$$

An oxidant (resp. reductant) in the solution can take (resp. give) an electron to the electrode to become a reductant (resp. oxidant). The most common example of such a reversible redox couple in thermogalvanic cell is that of Fe${\left(\mathrm{CN}\right)}_{6}^{3-}$/Fe${\left(\mathrm{CN}\right)}_{6}^{4-}$ in aqueous media [2,8,27,28,29].

The (electro)chemical potential (${\tilde{\mu }}_{j}$) equivalent of Equation (2) at the local thermodynamic equilibrium near the electrode is:

(5) $$\sum_{j}\nu_{j}{\tilde{\mu }}_{j}+{\tilde{\mu }}_{e-}=0$$

with

(6) $${\tilde{\mu }}_{j}=\mu_{j}+z_{j}eV^{i}$$

and

(7) $${\tilde{\mu }}_{e^{-}}=\mu_{e^{-}}-eV^{electrode}$$

where $V^{i}$ the internal potential of the solution near the electrode and $V^{electrode}$ the potential inside the conducting electrode. Then the potential difference between two electrodes held at different temperatures can be written as:

(8)eΔVelectrode=∑jνjΔμj+zjeΔVi+Δμe−(9)=∑jνjΔμj+ΔVi·e·∑jνjzj+Δμe−(10)=ΔΔrG+ΔVi·e·∑jνjzj+Δμe−

where $\Delta_{r}G=\sum_{j}\nu_{j}\mu_{j}$ is the Gibbs free energy of the redox half reaction. The conservation of charge imposed by Equation (2): i.e., $\sum_{j}\lambda_{j}z_{j}=1$ and the definition of internal electric potential; i.e., $\Delta V^{i}=-{\mathrm{Se}}_{\mathrm{int}}\cdot \Delta T$ simplifies the above expression to:

(11)eΔVelectrode=ΔΔrG−e·Seint·ΔT+Δμe−(12)Se=−ΔVelectrodeΔT=−1e·ΔTΔΔrG+Δμe−+Seint

We suppose that the chemical potential difference of electrons between the two conducting electrodes ($\Delta \mu_{e^{-}}$) is negligible, i.e., the Seebeck coefficient of the metal (of the order of µV/K) is negligibly small compared to that of the solution (of the order of mV/K). Therefore:

(13) $$\mathrm{Se}={\mathrm{Se}}_{\mathrm{int}}-\frac{\Delta \Delta_{r}G}{e\cdot \Delta T}$$

The measured Seebeck coefficient at the electrodes is thus the sum of two terms with distinct origins: the internal Seebeck coefficient created by the ensemble of ions/particles in the solution and the term due to the redox couple.

The internal electric field ${\vec{E}}_{\mathrm{int}}$ is the field experienced by one charged particle in the bulk of the solution. It is of fundamental importance for a large number of diffusion phenomena of charged species in electrolytes [17,18,19,20,30,31,32,33]. However, ${\vec{E}}_{\mathrm{int}}$ is very difficult to measure experimentally, given the fact that the introduction of metallic electrodes inevitably induces surface phenomena (e.g., electronic double layer formation). The corresponding internal Seebeck coefficient is given by:

(14) $${\vec{E}}_{\mathrm{int}}={\mathrm{Se}}_{\mathrm{int}}\vec{\nabla }T$$

where ${\vec{E}}_{\mathrm{int}}$ can be obtained from the particle current ${\vec{J}}_{N_{i}}$ equation,

(15) $${\vec{J}}_{N_{i}}=-D_{i}\left[\vec{\nabla }n_{i}+n_{i}\frac{{\hat{S}}_{i}}{k_{B}T}\vec{\nabla }T-n_{i}\frac{\xi_{i}e}{k_{B}T}\vec{E}\right]$$

We now show the derivation of this particle current Equation (15) and the dependence of the parameters $D_{i}$, ${\hat{S}}_{i}$ and $\xi_{i}$ on physical quantities such as particle concentration and magnetic field.

## 3. Particle Flux

In this section, we attempt to establish the particle flux Equation (15) with explicit expressions for $D_{i}$, ${\hat{S}}_{i}$ and $\xi_{i}$ as a function of physical quantities. As it has been done in many previous works, we start from Onsager’s theorem applied to liquid electrolytes. The three-dimensional system (liquid inside a thermogalvanic cell) considered here is composed of *p* species of mobile particles. The system is considered to be weakly out-of-equilibrium, such that thermodynamic quantities (*T*, *P*, *V*, $N_{i}$, ...) vary slowly with respect to the system size, and thus one can define a local, mesoscopic equilibrium at all points $\vec{r}$ in the system by $T(\vec{r})$, $P(\vec{r})$, etc. The energy flux $\vec{J_{U}}(\vec{r})$ represents the quantity of energy passing through a unit surface area per unit time in J·s${}^{-1}\cdot$m${}^{-2}$ and the particle flux ${\vec{J}}_{Ni}(\vec{r})$, i∈〚1;p〛, the number of particles of ith species crossing through a unit surface area per unit time, in s${}^{-1}\cdot$m${}^{-2}$.

In the framework of Onsager’s theorem [34,35] built upon the particle and energy conservation laws, the energy and the particle flux are proportional to the gradients of their respective conjugated variables obtained from the differential entropy of the system (to a first approximation):

(16) $$dS=\frac{dU}{T}+\frac{P}{T}\cdot dV-\sum_{i=1}^{p}\frac{{\tilde{\mu }}_{i}}{T}\cdot dN_{i}$$

which gives:

(17) $$\frac{\partial S\left(U,V,N_{1},\ldots ,N_{p}\right)}{\partial U}=\frac{1}{T}$$

(18) $$\frac{\partial S\left(U,V,N_{1},\ldots ,N_{p}\right)}{\partial V}=\frac{P}{T}$$

(19) $$\frac{\partial S\left(U,V,N_{1},\ldots ,N_{p}\right)}{\partial N_{i}}=-\frac{{\tilde{\mu }}_{i}}{T}$$

with *U*, the total energy of the system, *V*, the volume and *N*, the number of particles of ith specie. In the presence of magnetic particles, the magnetic energy should be added to *U*, i.e., $U+U^{H}$ where $U^{H}=-\mu_{0}\cdot M_{i}\cdot H$ with $M_{i}$ the magnetization of ith species and *H* the magnetic field. ${\tilde{\mu }}_{i}$ is the chemical potential which is composed of three terms in the general case:

(20) $${\tilde{\mu }}_{i}={\left(\frac{\partial F}{\partial N_{i}}\right)}_{T,V,H}=\underset{\mathrm{chemical}\;\mathrm{component}}{\underset{︸}{\mu_{i}}}+\underset{\mathrm{electric}\;\mathrm{component}}{\underset{︸}{\mu_{i}^{e}}}+\underset{\mathrm{magnetic}\;\mathrm{component}}{\underset{︸}{\mu_{i}^{H}}}$$

*F* is the free energy of the system (F=U−TS). The electric component is null for neutral particles and the magnetic component is negligible for diamagnetic and for most paramagnetic particles. $\mu_{i}+\mu_{i}^{e}$ is often referred to as the electrochemical potential.

Then the energy and the particle flux of a system consisting of particles of *p* species is written as:

(21) $$\left(\begin{array}{c} {\vec{J}}_{U}\\ {\vec{J}}_{N_{1}}\\ {\vec{J}}_{N_{2}}\\ \vdots \\ {\vec{J}}_{N_{p}} \end{array}\right)=\left[\begin{array}{ccccc} L_{UU} & L_{U1} & L_{U2} & \cdots & L_{Up}\\ L_{U1} & L_{11} & 0 & \cdots & 0\\ L_{U2} & 0 & L_{22} & \cdots & 0\\ \vdots & \vdots & \vdots & \ddots & \vdots \\ L_{Up} & 0 & 0 & \cdots & L_{pp} \end{array}\right]\left(\begin{array}{c} \vec{\nabla }\left(\frac{1}{T}\right)\\ -\vec{\nabla }\left(\frac{{\tilde{\mu }}_{1}}{T}\right)\\ -\vec{\nabla }\left(\frac{{\tilde{\mu }}_{2}}{T}\right)\\ \vdots \\ -\vec{\nabla }\left(\frac{{\tilde{\mu }}_{p}}{T}\right) \end{array}\right)$$

Here, we have used the Onsager reciprocity relations [34]: $\forall i,\forall j,\;L_{ij}=L_{ji}$ and supposed that the terms $L_{ij}\vec{\nabla }\left(\frac{{\tilde{\mu }}_{j}}{T}\right)$ are negligibly small compared to the terms $L_{ii}\vec{\nabla }\left(\frac{{\tilde{\mu }}_{i}}{T}\right)$, for all j≠i. Thus we have p+1 vector equations:

(22)J→U=LUU·∇→1T−∑i=1pLUi·∇→μ˜iT(23)∀i∈〚1;p〛,J→Ni=LUi·∇→1T−Lii·∇→μ˜iT

By combining (23) and (21) the energy flux can be expressed simply as:

(24) $${\vec{J}}_{U}=\left[L_{UU}-\sum_{i=1}^{p}\frac{{L_{Ui}}^{2}}{L_{ii}}\right]\cdot \vec{\nabla }\left(\frac{1}{T}\right)+\sum_{i=1}^{p}\frac{L_{Ui}}{L_{ii}}{\vec{J}}_{N_{i}}$$

The heat flux ${\vec{J}}_{Q}$ is the difference between the total energy flux ${\vec{J}}_{U}$ and ${\tilde{\mu }}_{i}\cdot {\vec{J}}_{N_{i}}$ (energy carried by particles):

(25) $${\vec{J}}_{Q}={\vec{J}}_{U}-\sum_{i=1}^{p}{\tilde{\mu }}_{i}\cdot {\vec{J}}_{N_{i}}=\sum_{i=1}^{p}{\bar{\bar{Q}}}_{i}{\vec{J}}_{N_{i}}-\kappa \vec{\nabla }T$$

where

(26)Q¯¯i=LUiLii−μ˜i(27)κ=1T2LUU−∑i=1pLUi2Lii

${\bar{\bar{Q}}}_{i}$ is the quantity of transported heat by one particle *i* moving in the system, which is different from ${\tilde{\mu }}_{i}$. This transported heat is associated to the transported entropy ${\bar{\bar{S}}}_{i}$ :

(28) $${\bar{\bar{S}}}_{i}=\frac{{\bar{\bar{Q}}}_{i}}{T}$$

κ is the thermal conductivity of the system, and we remark that in the absence of particle flux; i.e., $\forall i,{\vec{J}}_{N_{i}}=\vec{0}$, one recovers Fourier’s law:

(29) $${\vec{J}}_{Q}=-\kappa \vec{\nabla }T$$

In addition, finally, ∀i∈〚1;p〛, the particle flux can be expressed in terms of ${\bar{\bar{S}}}_{i}$ as

(30) $${\vec{J}}_{N_{i}}=-\frac{L_{ii}}{T}\left[\vec{\nabla }{\tilde{\mu }}_{i}+{\bar{\bar{S}}}_{i}\cdot \vec{\nabla }T\right]$$

The particle flux has thus two components; a term related to the chemical potential gradient, e.g., the concentration gradient, and the entropic term related to the temperature gradient. In order to express this flux, it is thus necessary to know the expression for three types of chemical potential (20) of all particles/ions ${\tilde{\mu }}_{i}$.

### 3.1. Chemical Potential of Magnetic Nanofluids

In magnetic nanofluids, two types of particles are involved

- Diamagnetic solutes, ions or neutral species, all less than nanometer in size. These solutes will be treated as an ideal gas.
- Charged magnetic particles whose characteristic sizes are in the order of ten nanometers. These particles will be described by an effective hard-sphere model derived from Carnahan-Starling equation of state and the inter-particle magnetic interactions are taken into account through a mean-field approach.

The chemical potential can be defined from several thermodynamic state functions. We adopt here the definition obtained from the free energy of the system *F*:

(31) $$\tilde{\mu }={\left(\frac{\partial F}{\partial N}\right)}_{T,V,H}={\left(\frac{\partial f}{\partial n}\right)}_{T,H}$$

where *N* is the number of particles in the system, $f=\frac{F}{V}$ the free energy per unit volume and $n=\frac{N}{V}$ the number density of the particles.

Free chemical energy, and thus the chemical potential of a system composed of particles can be obtained from its equation of state, noting that the differential of the free energy is:

(32) $$dF=-PdV-SdT+\sum_{i=1}^{p}\mu_{i}dN_{i}$$

and

(33) $${\left(\frac{\partial F}{\partial V}\right)}_{T,N_{i},H}=-P$$

Note that we do not use the Gibbs-Duhem thermodynamic equation which is convenient for describing thermodiffusive [20,36] systems where one takes in account the change in the local pressure upon $i_{th}$ specie due to the change in the chemical potential of other (k≠i) species. Here we assume that such force, arising from the cross-terms in the Onsager equations is negligibly small.

Two types of equations of states are possible.

Case I: ideal gas

The state equation of a perfect gas at equilibrium, i.e., composed of point-like non-interacting particles, is well known:

(34) $$P\cdot V=N\cdot k_{B}\cdot T$$

with *P*, the pressure, *V*, the volume, *N*, the number of particles, $k_{B}$ the Boltzmann constant and *T*, the temperature. This state equation is suitable for diluted gas for which the particle size is much smaller than the average distance between them and the inter-particle interactions are negligible.

Case II: Hard-sphere gas

A generic equation of state for a non-ideal gas can be obtained through a series function (of particle density), known as a virial expansion:

(35) $$\frac{P}{k_{B}\cdot T}=\frac{N}{V}+\sum_{i=2}^{\infty }B_{i}{\left(\frac{N}{V}\right)}^{i}=\frac{N}{V}+B_{2}{\left(\frac{N}{V}\right)}^{2}+B_{3}{\left(\frac{N}{V}\right)}^{3}+B_{4}{\left(\frac{N}{V}\right)}^{4}+\cdots$$

$B_{i}$ are called *i^th^ virial coefficients* that depend on temperature. For $B_{1}=1$ and up to the 1st order in $\frac{N}{V}$, one recovers the equation of state of an ideal gas.

Nanofluids can be approximated to a hard-sphere gas, consisting of spherical particles with a finite diameter. In this case, the particle density can be converted to the volume fraction ϕ (always less than one)

(36) $$\phi =\frac{V_{part}}{V}=\frac{N\cdot \pi \cdot d^{3}}{6\cdot V}=n\cdot v_{0}$$

with $V_{part}$ the total volume occupied by the particles in the system, and $v_{0}$ the volume of one particle. Carnahan and Starling [37] remarked that under such condition the virial coefficients are very close to those of a power series; i.e.,

(37) $$S_{CS}\left(\phi \right)=1+\sum_{n=2}^{\infty }\left(n^{2}+n-2\right)\phi^{n-1}$$

The series converges for |ϕ|<1, which is always the case as ϕ is the volume fraction of particles and the Carnahan-Starling equation of state for hard-sphere gas is expressed as:

(38) $$\frac{P\cdot V}{N\cdot k_{B}\cdot T}=\frac{1+\phi +\phi^{2}-\phi^{3}}{{\left(1-\phi \right)}^{3}}$$

Indeed, the pressure described above corresponds to that of *osmotic* pressure rather than the real pressure of the liquid. An important quantity in a Carnahan-Starling hard-sphere gas is then the *isothermal osmotic compressibility*, χ:

(39) $$\chi =\frac{k_{B}T}{v_{0}}{\left(\frac{\partial P}{\partial \phi }\right)}^{-1}$$

χ describes the change in pressure due to the variation of particle concentration, and in the case of a Carnahan-Starling gas $\chi_{CS}$ is:

(40) $$\chi_{CS}\left(\phi \right)=\frac{{\left(1-\phi \right)}^{4}}{1+4\phi +4\phi^{2}-4\phi^{3}+\phi^{4}}$$

The free chemical energy can be calculated once again, for an ideal gas and a hard-sphere gas cases separately.

Case I: Ideal gas

Starting from Equation (34), the free energy can then be obtained from Sackur–Tetrode equation:

(41) $$F_{id}=N\cdot f_{0}\left(T\right)+N\cdot k_{B}\cdot T\cdot \ln\left(n\right)$$

with $f_{0}$ a function of temperature *T* associated to the internal degrees of freedom of the particles and constant with respect to the number of particles *N*. Equivalently, one can express $F_{id}$ as a function of ϕ rather than *n* :

(42) $$F_{id}=N\cdot f_{0}\left(T\right)+N\cdot k_{B}\cdot T\cdot \ln\left(\frac{\phi }{v_{0}}\right)$$

Case II: Hard-sphere gas

For a hard sphere gas obeying the Carnahan-Starling equation of state, the pressure is expressed as:

(43) $$P_{CS}=\frac{N\cdot k_{B}\cdot T}{V}\cdot \frac{1+\phi +\phi^{2}-\phi^{3}}{{\left(1-\phi \right)}^{3}}=-{\left(\frac{\partial F_{CS}}{\partial V}\right)}_{T,N}$$

By noting that:

(44) $${\left(\frac{\partial V}{\partial \phi }\right)}_{N}=-\frac{v_{0}}{\phi^{2}},$$

one obtains:

(45) $$\begin{array}{cc} \frac{1}{N\cdot k_{B}\cdot T}{\left(\frac{\partial F_{CS}}{\partial \phi }\right)}_{T,N} & =\frac{1+\phi +\phi^{2}-\phi^{3}}{\phi \cdot {\left(1-\phi \right)}^{3}}. \end{array}$$

The excess free energy of a hard-sphere gas can now be calculated from subtracting the ideal case from Equation (45)

(46) $$\begin{array}{cc} \frac{1}{N\cdot k_{B}\cdot T}\left[{\left(\frac{\partial F_{CS}}{\partial \phi }\right)}_{T,N}-{\left(\frac{\partial F_{id}}{\partial \phi }\right)}_{T,N}\right] & =\frac{4-2\cdot \phi }{{\left(1-\phi \right)}^{3}} \end{array}$$

Noting that:

(47) $$\frac{d}{dx}\left[\frac{4x-3x^{2}}{{\left(1-x\right)}^{2}}\right]=\frac{4-2x}{{\left(1-x\right)}^{3}}$$

the above expression is simplified to:

(48) $$F_{CS}-F_{id}=N\cdot k_{B}\cdot T\cdot \frac{4\phi -3\phi^{2}}{{\left(1-\phi \right)}^{2}}$$

The integration constant, which is a function of *T* and *N*, is included in the constant $N\cdot f_{0}\left(T\right)$ that appears in $F_{id}$. The free energy of a hard-sphere gas is then expressed as:

(49) $$F_{CS}=N\cdot f_{0}\left(T\right)+N\cdot k_{B}\cdot T\cdot \ln\left(\frac{\phi }{v_{0}}\right)+N\cdot k_{B}\cdot T\cdot \frac{4\phi -3\phi^{2}}{{\left(1-\phi \right)}^{2}}$$

or, equivalently:

(50) $$f_{CS}=\frac{\phi }{v_{0}}\cdot f_{0}\left(T\right)+\frac{\phi }{v_{0}}\cdot k_{B}\cdot T\cdot \ln\left(\frac{\phi }{v_{0}}\right)+\frac{\phi }{v_{0}}\cdot k_{B}\cdot T\cdot \frac{4\phi -3\phi^{2}}{{\left(1-\phi \right)}^{2}}$$

Thus we recover the first two terms of an ideal gas, while the last term corresponds to the correction for a hard-sphere case.

Combining Equations (50) and (31) (via (47)), we can now obtain the expression for the chemical potential μ of a hard-sphere gas:

(51)μv0=1v0·∂fCS∂n=∂fCS∂ϕ(52)=f0(T)v0+kB·Tv0+kB·Tv0·lnϕv0+kB·Tv0·8ϕ−9ϕ2+3ϕ3(1−ϕ)3

Then, introducing $\mu^{\circ }$ as:

(53) $$\mu^{\circ }\left(T\right)=f_{0}\left(T\right)+k_{B}\cdot T$$

one obtains:

(54) $$\mu =\mu^{\circ }\left(T\right)+k_{B}\cdot T\cdot \ln\left(\frac{\phi }{v_{0}}\right)+k_{B}\cdot T\cdot \frac{8\phi -9\phi^{2}+3\phi^{3}}{{\left(1-\phi \right)}^{3}}$$

Note that the chemical potential of a hard sphere gas is equal to that of an ideal gas when the sphere radius approaches 0; that is, ϕ→ 0, at a constant $n=\phi /v_{0}$.

### 3.2. Electric Component $\mu^{e}$

We consider now a particle with an electric charge z·e under an electrical potential $V(\vec{r})$. The electric component of the chemical potential is simply the potential energy of the particle up to a constant:

(55) $$\mu^{e}=z\cdot e\cdot V\left(\vec{r}\right)$$

Then, the associated free energy is:

(56) $$F^{e}=N\cdot z\cdot e\cdot V\left(\vec{r}\right)$$

Equivalently,

(57) $$f^{e}=\frac{\phi }{v_{0}}\cdot z\cdot e\cdot V\left(\vec{r}\right)$$

The above equation is valid for a point-like species. In the case of nanoparticles, the charge number *z* must be replaced by the effective charge number $\xi^{0}$. The electric component of the chemical potential and of the free energy become:

(58)μe=ξ0·e·Vr→(59)fe=ϕv0·ξ0·e·Vr→

### 3.3. Magnetic Component $\mu^{H}$

In the case of magnetic nanofluids, the charged particles are made of ferro- or ferri-magnetic material and thus the magnetic component $\mu^{H}$ needs to be taken in account. We assume that in a liquid medium, these particles can move and rotate freely such that the magnetic anisotropy energy can be neglected. Furthermore, they are sufficiently small and the temperature sufficiently high and thus are in the superparamagnetic state.

#### 3.3.1. Single Particle Magnetization

**Figure 2:**
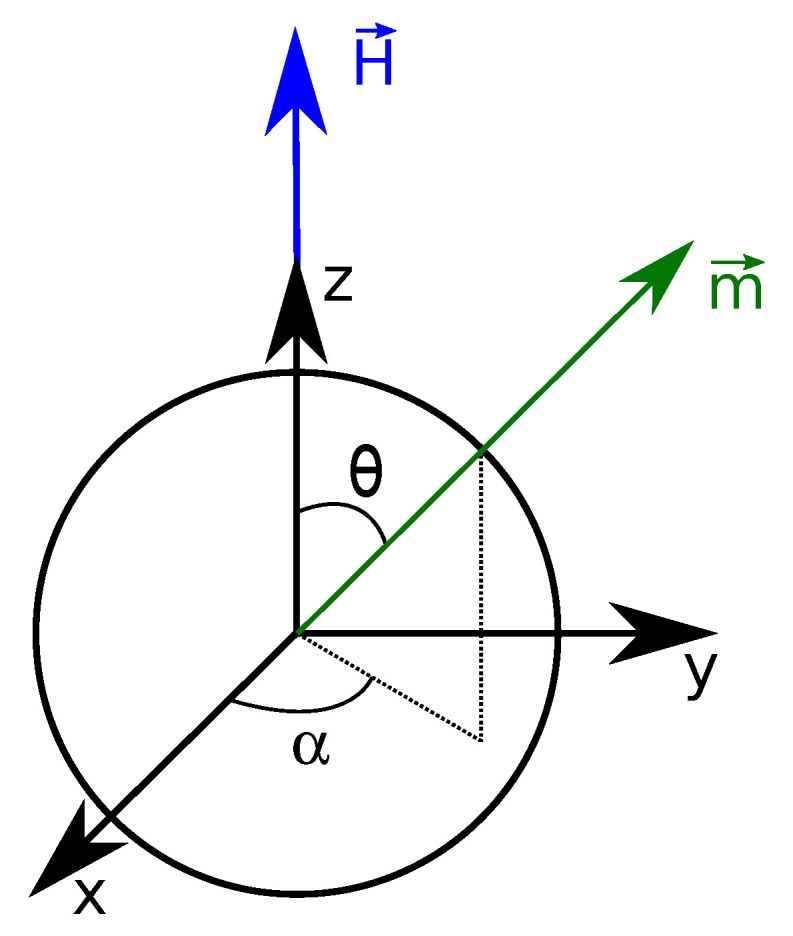
Single magnetic nanoparticle. The magnetic field $\vec{H}$ is applied along the *z*-axis. θ defines the angle between $\vec{m}$ and the unit vector along z, ${\vec{u}}_{z}$, and α the angle between the projection of $\vec{m}$ in the xy plane and the unit vector along *x*, ${\vec{u}}_{x}$.

We first consider a very dilute system in which magnetic nanoparticles do not interact with one another. The magnetization $\vec{M}$ of an assembly of non-interacting particles is solely due to the orientation of the magnetic moment $\vec{m}$ of individual particles along the applied magnetic field $\vec{H}$. As shown in Figure 2 $\vec{H}=H{\vec{u}}_{z}$ and thus the *x* and *y* components of magnetization averaged out to zero by symmetry around the *z*-axis. Therefore $\vec{M}$ can be written as:

(60) $$\vec{M}=M{\vec{u}}_{z}$$

The magnetic energy of a single nanoparticle is:

(61) $$\begin{array}{cc} U^{H} & =-\mu_{0}\cdot \vec{m}\cdot \vec{H}=-\mu_{0}\cdot m\cdot H\cdot \cos\left(\theta \right) \end{array}$$

where $\mu_{0}$ is the magnetic permeability of vacuum and θ, the angle between the vectors $\vec{m}$ and ${\vec{u}}_{z}$. At a thermodynamic equilibrium, the particles are distributed according to the Boltzmann statistics and the magnetization is given by the well-known Langevin function [38]:

(62) $$M=n\cdot m\cdot L\left(\xi \right)\;\;\;\mathrm{with}\;\xi =\frac{\mu_{0}\cdot m\cdot H}{k_{B}\cdot T}$$

where ξ is the Langevin parameter and $L\left(x\right)=\coth\left(x\right)-\frac{1}{x}$ the Langevin function. The magnetization of particles is thus zero on average when ξ→0, that is, the thermal energy ($k_{B}T$) is much larger than the magnetic energy ($\mu_{0}mH$). On the contrary, i.e., for ξ→∞ ($k_{B}T\ll \mu_{0}mH$), all particles’ magnetic moments are aligned parallel to the applied field direction and the magnetization reaches its saturation value n·m.

#### 3.3.2. Magnetization of Interacting Particles: Mean-Field Approach

By increasing the particle concentration, not only the applied magnetic field but also that exerted by its neighboring particles starts to influence the magnetization of the assembly (dipole-dipole interactions). In the framework of mean field theory, the effective magnetic field felt by a single particle ${\vec{H}}_{e}$ is expressed by [39]:

(63) $${\vec{H}}_{e}=\overset{\mathrm{macroscopic}\;\mathrm{magnetic}\;\mathrm{field}}{\overset{︷}{\vec{H}}}+\overset{\mathrm{local}\;\mathrm{field}}{\overset{︷}{\lambda \cdot \vec{M}}}$$

The first term corresponds to the macroscopic magnetic field, which is the sum of two terms: ${\vec{H}}_{0}$ is the uniformly applied external field and ${\vec{H}}_{1}$, the internal demagnetization field created by magnetic moments of other particles. The second term is proportional to the local magnetization of the system and it represents the influence of nearby particles. λ is a non-dimensional proportionality coefficient, which is zero in the absence of inter-particle magnetic interactions, and typically $\frac{1}{3}$ in a uniformly magnetized medium [38]. In the case of aqueous ferrofluids, this value has been determined experimentally [40,41,42,43,44] and numerically [45,46] to be λ=0,22.

This effective magnetic field must satisfy the self-consistency equation:

(64) $$\xi_{e}=\frac{\mu_{0}\cdot m\cdot H_{e}}{k_{B}\cdot T}=\underset{\xi_{0}}{\underset{︸}{\frac{\mu_{0}\cdot m\cdot H}{k_{B}\cdot T}}}+\frac{\mu_{0}\cdot m\cdot \lambda \cdot M}{k_{B}\cdot T}$$

Substituting *M* by Equation (62):

(65) $$\xi_{e}=\xi_{0}+\frac{\mu_{0}\cdot \lambda \cdot n\cdot m^{2}\cdot L\left(\xi_{e}\right)}{k_{B}\cdot T}=\xi_{0}+\lambda \psi_{dd}\phi \cdot L\left(\xi_{e}\right)$$

with

(66) $$\psi_{dd}=\frac{\mu_{0}m^{2}}{v_{0}k_{B}T}$$

where $\psi_{dd}$ is the dipolar interaction parameter representing the ratio between the dipole-dipole interaction energy (i.e., inter-particle distance is equal to the particle diameter) and the thermal energy.

The total magnetic energy (per unit volume) of the system is composed of two terms :

(67)UH=−μ0·N·<m→>H→−μ0·N·<m→>·λ·M→2(68)=−μ0·V·M·H−μ0·λ·V·M22

The first term corresponds to the magnetic energy associated to the direct interaction between the magnetic moments *m* and the applied field $\vec{H}$ while the second term corresponds to the pair interaction between the magnetic moments themselves. A factor $\frac{1}{2}$ appears to avoid double-counting the same particle combinations.

The magnetic component of entropy can be calculated using the Shannon formula [39]:

(69) $$s^{H}=-n\cdot \int_{\theta =0}^{\pi }k_{B}\cdot P\left(\theta \right)\cdot \ln\left(P(\theta )\right)\cdot 2\pi \cdot \sin\left(\theta \right)d\theta$$

which gives

(70) $$\begin{array}{cc} \frac{s^{H}}{n\cdot k_{B}} & =\ln\left(\frac{\xi_{e}}{2\pi \left(e^{\xi_{e}}-e^{-\xi_{e}}\right)}\right)+\xi_{e}\cdot L\left(\xi_{e}\right) \end{array}$$

In addition, using Equations (62) and (65):

(71)n·kB·ξe·Lξe=μ0·n·m·H·LξeT+μ0·n2·m2·λ·L2ξeT(72)=μ0·M·HT+μ0·λ·M2T

By combining Equations (67), (70) and (72), one can now calculate the magnetic free energy as:

(73) $$f^{H}=n\cdot k_{B}\cdot T\cdot \ln\left(\frac{\xi_{e}}{2\pi \left(e^{\xi_{e}}-e^{-\xi_{e}}\right)}\right)+\frac{\mu_{0}\cdot \lambda \cdot M^{2}}{2}$$

In terms of $v_{0}$ and ϕ,

(74) $$f^{H}=\frac{\phi }{v_{0}}\cdot k_{B}\cdot T\cdot \ln\left(\frac{\xi_{e}}{2\pi \left(e^{\xi_{e}}-e^{-\xi_{e}}\right)}\right)+\frac{\mu_{0}\cdot \lambda \cdot \phi^{2}\cdot m^{2}\cdot L^{2}\left(\xi_{e}\right)}{2\cdot v_{0}^{2}}$$

#### 3.3.3. Expression for $\mu^{H}$

The magnetic component of the chemical potential can be deduced from the free energy:

(75)μHv0=∂fH∂ϕ(75)=kBTv0lnξe2π·eξe−e−ξe−ϕLξe∂ξe∂ϕ+μ0λm2v02ϕL2ξe+ϕ2LξeL′ξe∂ξe∂ϕ

where $\frac{\partial \xi_{e}}{\partial \phi }$ is determined from the self-consistency equation (Equation (65)):

(77)∂ξe∂ϕ=μ0λm2v0kBTLξe+λψddϕL′ξe∂ξe∂ϕ(78)=μ0λm2v0kBT1−λψddϕL′ξeLξe

Then the final expression of $\mu^{H}$ is simplified to:

(79) $$\mu^{H}=k_{B}T\ln\left(\frac{\xi_{e}}{4\cdot \pi \sinh(\xi_{e})}\right)$$

The magnetic part of the chemical potential is thus a monotonous and negative function, with its minimum at $\xi_{e}\to 0$. The higher $\xi_{e}$ is, the larger the average magnetic energy of one particle and the probability of its magnetic moment being aligned in the direction of magnetic field become.

### 3.4. Total Chemical Potential

The chemical potential of one charged colloidal and superparamagnetic particle can now be obtained by summing the three components; chemical (Equation (54)), electric (Equation (55)) and magnetic (Equation (79)).

(80) $$\tilde{\mu }=\mu^{\circ }+k_{B}T\ln\left(\frac{\phi }{v_{0}}\right)+k_{B}T\frac{8\phi -9\phi^{2}+3\phi^{3}}{{\left(1-\phi \right)}^{3}}+z\cdot e\cdot V+k_{B}T\ln\left(\frac{\xi_{e}}{4\pi \cdot \sinh(\xi_{e})}\right)$$

Again, we perform transformation of $f_{0}$, the free energy term associated with the internal degrees of freedom the particle; $f_{0}\left(T\right)-k_{B}T\ln\left(4\pi \right)\to f_{0}\left(T\right)$, to obtain the final expression:

(81) $$\tilde{\mu }=\mu^{\circ }+k_{B}T\ln\left(\frac{\phi }{v_{0}}\right)+k_{B}T\frac{8\phi -9\phi^{2}+3\phi^{3}}{{\left(1-\phi \right)}^{3}}+z\cdot e\cdot V+k_{B}T\ln\left(\frac{\xi_{e}}{\sinh(\xi_{e})}\right)$$

In the case of point-like and non-magnetic particles (i.e.,majority of ions found in common electrolytes), one recovers the classic expression electrochemical potential;

(82) $$\tilde{\mu }=\mu^{\circ }+k_{B}T\ln\left(n\right)+z\cdot e\cdot V$$

with *n* the particle concentration.

### 3.5. General Expression for Particle Flux

We can now determine the expression for particle flux (Equation (30)) of charged and magnetic colloidal particles by calculating the gradient of the magneto-electrochemical potentials ($\vec{\nabla }{\tilde{\mu }}_{i}$).

(83) $$\vec{\nabla }{\tilde{\mu }}_{i}={\left(\frac{\partial {\tilde{\mu }}_{i}}{\partial N_{i}}\right)}_{T,H}\vec{\nabla }N_{i}+{\left(\frac{\partial {\tilde{\mu }}_{i}}{\partial T}\right)}_{N_{i},H}\vec{\nabla }T+{\left(\frac{\partial {\tilde{\mu }}_{i}}{\partial H}\right)}_{N_{i},T}\left(\frac{\vec{H}}{\left|\vec{H}\right|}\cdot \vec{\nabla }\right)\vec{H}+\xi_{i}^{0}\cdot e\cdot \vec{\nabla }V$$

Here $\vec{H}={\vec{H}}_{0}+\delta \vec{H}$ stands for the total magnetic field (${\vec{H}}_{0}$ applied field and $\delta \vec{H}$ field perturbations).

Below, we first calculate the partial derivatives of ${\tilde{\mu }}_{i}$ to obtain the general expressions for particle flux. The variation in the local magnetic field will be treated later in two particular cases where the temperature gradient and the magnetic field are applied either perpendicularly or in parallel.

#### 3.5.1. Chemical Potential Gradient: With Respect to *N*

By definition, $\phi_{i}=v_{0}\frac{N_{i}}{V}$ and thus:

(84) $${\left(\frac{\partial {\tilde{\mu }}_{i}}{\partial N_{i}}\right)}_{T,H}\vec{\nabla }N_{i}={\left(\frac{\partial {\tilde{\mu }}_{i}}{\partial \phi_{i}}\right)}_{T,H}\vec{\nabla }\phi_{i}$$

then, according to (81) and using (78),

(85)1kBT∂μ˜i∂ϕiT,H=1ϕi+8−2ϕi1−ϕi4+sinh(ξe)ξe1sinh(ξe)−cosh(ξe)ξesinh2(ξe)∂ξe∂ϕi(86)=1ϕi+8−2ϕi1−ϕi4−μ0λm2v0kBT1−λψddϕiL′ξeL2ξe(87)=1ϕi+8−2ϕi1−ϕi4−αλϕi,Hϕi

with

(88) $$\alpha_{\lambda }\left(\phi_{i},H\right)=\frac{\lambda \psi_{dd}\phi_{i}}{1-\lambda \psi_{dd}\phi_{i}L^{\prime }\left(\xi_{e}\right)}L^{2}\left(\xi_{e}\right)$$

and thus:

(89) $$\begin{array}{cc} {\left(\frac{\partial {\tilde{\mu }}_{i}}{\partial \phi_{i}}\right)}_{T,H} & =k_{B}T\left(\frac{1+4\phi_{i}+4\phi_{i}^{2}-4\phi_{i}^{3}+\phi^{4}}{\phi_{i}{\left(1-\phi_{i}\right)}^{4}}-\frac{\alpha_{\lambda }\left(\phi_{i},H\right)}{\phi_{i}}\right) \end{array}$$

(90) $$\begin{array}{cc} {\left(\frac{\partial {\tilde{\mu }}_{i}}{\partial \phi_{i}}\right)}_{T,H} & =k_{B}T\left(\frac{1}{\chi_{CS}\left(\phi_{i}\right)\phi_{i}}-\frac{\alpha_{\lambda }\left(\phi_{i},H\right)}{\phi_{i}}\right) \end{array}$$

**Figure 3:**
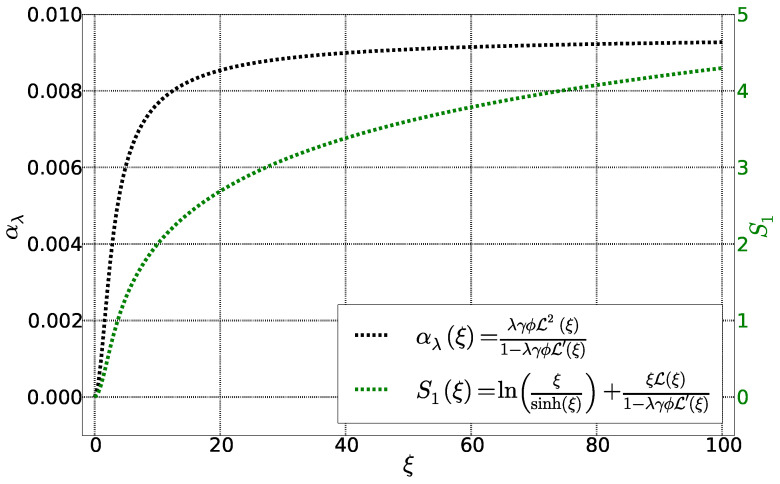
Fonctions $\alpha_{\lambda }$ and $S_{1}$ as a function of Langevin parameter ξ. The other parameters are fixed: ϕ = 0.01, λ = 0.22 et $\psi_{dd}$ = 4.3.

The first term corresponds to the hard-core interactions between the spherical particles in the Carnahan-Starling formalism and the second term due to to magnetic interactions (Figure 3). It is zero for particles without dipolar interactions (λ=0) as well as in the absence of applied magnetic field ($\xi_{e}=0$). In addition, it saturates once the magnetic energy is very large compared to the thermal energy, i.e., ξ→∞. This term exists, however, even for a homogeneous fluid, without a temperature gradient or concentration gradient, under a magnetic field.

#### 3.5.2. Derivative of ${\tilde{\mu }}_{i}$ with Respect to Temperature

The expression of the second term can be obtained from Equation (32).

(91) $$\frac{\partial {\tilde{\mu }}_{i}}{\partial T}=\frac{\partial \mu_{i}}{\partial T}+\frac{\partial \mu_{i}^{H}}{\partial T}$$

According to Schwartz’s theorem the first term becomes:

(92) $$\frac{\partial \mu_{i}}{\partial T}=\frac{\partial }{\partial T}\frac{\partial F}{\partial N_{i}}=\frac{\partial }{\partial N_{i}}\frac{\partial F}{\partial T}=-\frac{\partial S}{\partial N_{i}}=-S_{i}\left(\phi_{i},H\right)$$

where $S_{i}$ is the partial entropy of the ith species. The second term gives:

(93) $$\frac{\partial \mu_{i}^{H}}{\partial T}=k_{B}\ln\left(\frac{\xi_{e}}{\sinh(\xi_{e})}\right)-k_{B}TL\left(\xi_{e}\right)\frac{\partial \xi_{e}}{\partial T}$$

then using (65):

(94) $$\begin{array}{cc} \frac{\partial \xi_{e}}{\partial T} & =-\frac{1}{T}\frac{\xi_{e}}{1-\lambda \psi_{dd}\phi_{i}L^{\prime }\left(\xi_{e}\right)} \end{array}$$

that is:

(95) $$\frac{\partial \mu_{i}^{H}}{\partial T}=k_{B}\ln\left(\frac{\xi_{e}}{\sinh(\xi_{e})}\right)+k_{B}\frac{\xi_{e}\cdot L\left(\xi_{e}\right)}{1-\lambda \psi_{dd}\phi_{i}L^{\prime }\left(\xi_{e}\right)}=k_{B}\cdot S_{1}\left(\phi_{i},H\right)$$

with

(96) $$S_{1}\left(\phi_{i},H\right)=\ln\left(\frac{\xi_{e}}{\sinh(\xi_{e})}\right)+\frac{\xi_{e}\cdot L\left(\xi_{e}\right)}{1-\lambda \psi_{dd}\phi_{i}L^{\prime }\left(\xi_{e}\right)}$$

and finally:

(97) $${\left(\frac{\partial {\tilde{\mu }}_{i}}{\partial T}\right)}_{N_{i},H}=-S_{i}\left(\phi_{i},H\right)+k_{B}\cdot S_{1}\left(\phi_{i},H\right)$$

In the case of non-magnetic particles (ξ=0) or in the absence of applied magnetic field (H=0), one recovers the classic partial entropy.

#### 3.5.3. Derivative of ${\tilde{\mu }}_{i}$ with Respect to Magnetic Field

From Equation (81)

(98) $${\left(\frac{\partial {\tilde{\mu }}_{i}}{\partial H}\right)}_{T,N_{i}}=\frac{\partial k_{B}T\ln\left(\frac{\xi_{e}}{\sinh(\xi_{e})}\right)}{\partial H}=-k_{B}TL\left(\xi_{e}\right)\frac{\partial \xi_{e}}{\partial H}$$

where the partial derivative of the Langevin parameter $\xi_{e}$ (Equation (65)) with respect to *H* is:

(99) $$\frac{\partial \xi_{e}}{\partial H}=\frac{\mu_{0}m}{k_{B}T}+\lambda \psi_{dd}\phi_{i}L^{\prime }\left(\xi_{e}\right)\frac{\partial \xi_{e}}{\partial H}=\frac{\mu_{0}m}{k_{B}T\left(1-\lambda \psi_{dd}\phi_{i}L^{\prime }\left(\xi_{e}\right)\right)}$$

leading to:

(100) $${\left(\frac{\partial {\tilde{\mu }}_{i}}{\partial H}\right)}_{T,N_{i}}=-\frac{\mu_{0}mL\left(\xi_{e}\right)}{1-\lambda \psi_{dd}\phi_{i}L^{\prime }\left(\xi_{e}\right)}$$

#### 3.5.4. Electric Term of the Chemical Potential Gradient

By definition, $\vec{\nabla }V=-\vec{E}$ with $\vec{E}$ the electric field. Therefore, electric term is re-written as:

(101) $$\xi_{i}^{0}\cdot e\vec{\nabla }V=-\xi_{i}^{0}\cdot e\vec{E}$$

#### 3.5.5. General Expressions for Chemical Potential Gradient and Particle Flux

The general expression for $\vec{\nabla }{\tilde{\mu }}_{i}$ can now be expressed by combining the above results.

(102) $$\begin{array}{cc} \vec{\nabla }{\tilde{\mu }}_{i}= & k_{B}T\left(\frac{1+4\phi_{i}+4\phi_{i}^{2}-4\phi_{i}^{3}+\phi_{i}^{4}}{\phi_{i}{\left(1-\phi_{i}\right)}^{4}}-\frac{\alpha_{\lambda }\left(\phi_{i},H\right)}{\phi_{i}}\right)\vec{\nabla }\phi_{i}+\left(-S_{i}\left(\phi_{i},H\right)+k_{B}S_{1}\left(\phi_{i},H\right)\right)\vec{\nabla }T-\\ & \frac{\mu_{0}mL\left(\xi_{e}\right)}{1-\lambda \psi_{dd}\phi_{i}L^{\prime }\left(\xi_{e}\right)}\left(\frac{\vec{H}}{\left|\vec{H}\right|}\cdot \vec{\nabla }\right)\vec{H}-\xi_{i}^{0}e\cdot \vec{E} \end{array}$$

Then the particle flux (Equation (30)) becomes:

(103) $$\begin{array}{cc} {\vec{J}}_{N_{i}}= & -D_{i}\left(\phi_{i}\right)[\left(\frac{1}{\chi_{CS}\left(\phi_{i}\right)}-\alpha_{\lambda }\left(\phi_{i},H\right)\right)\vec{\nabla }\frac{\phi_{i}}{v_{0}}+\frac{\phi_{i}}{v_{0}}\frac{{\hat{S}}_{i}^{0}\left(\phi_{i},H\right)+k_{B}S_{1}\left(\phi_{i},H\right)}{k_{B}T}\vec{\nabla }T-\\ & \frac{\phi_{i}}{v_{0}k_{B}T}\frac{\mu_{0}mL\left(\xi_{e}\right)}{1-\lambda \psi_{dd}\phi_{i}L^{\prime }\left(\xi_{e}\right)}\left(\frac{\vec{H}}{\left|\vec{H}\right|}\cdot \vec{\nabla }\right)\vec{H}-\frac{\xi_{i}^{0}e\cdot \phi_{i}}{v_{0}k_{B}T}\vec{E}] \end{array}$$

with

(104) $$\begin{array}{c} D_{i}\left(\phi_{i}\right)=k_{B}\cdot v_{0}\frac{L_{ii}}{\phi_{i}}=D_{i}^{0}\frac{\zeta^{0}}{\zeta (\phi_{i})} \end{array}$$

(105) $$\begin{array}{c} {\hat{S}}_{i}^{0}\left(\phi ,H\right)={\bar{\bar{S}}}_{i}-S_{i}\left(\phi ,H\right) \end{array}$$

$D_{i}\left(\phi_{i}\right)$ is the diffusion coefficient that takes in account the friction between the particle *i* and the surrounding liquid, ζ. $\zeta^{0}=6\pi \eta R_{H,i}$ is the friction at $\phi_{i}=0$ with η the viscosity of the liquid and $R_{H,i}$ the hydrodynamic radius of the particle *i*. Note that the friction of interacting system, ζ(ϕ) is not well known and also that here, a term dependent on $\left(dP/dT\right)\vec{\nabla }T$ does not appear unlike in the models developed by [20,47]. This is due to the assumption introduced in Section 3 that $L_{ij}\vec{\nabla }\left(\frac{{\tilde{\mu }}_{j}}{T}\right)<<L_{ii}\vec{\nabla }\left(\frac{{\tilde{\mu }}_{i}}{T}\right)$∀j≠i, i.e., that the variation of species j≠i chemical potential has little direct influence on the flux of *i* (Theoretically, however, the variation in species *j*’s chemical potential will modify the osmotic pressure locally and therefore influence the flux of all other species). For point-like particles, in the absence of temperature gradient, magnetic field and electric field, Equation (103) is simplified to the well-known Fick’s law :

(106) $${\vec{J}}_{N_{i}}=-D_{i}\left(\phi_{i}\right)\vec{\nabla }n_{i}$$

In addition, ${\hat{S}}_{i}^{0}$ is the Eastman Entropy of transfer as defined by de Groot [48] and Agar [49] (and not by Eastman [50]). This entropy corresponds to the difference between the transported entropy and the partial entropy of the particles, thus associated to the enthalpy ${\hat{h}}_{i}$ such that:

(107) $${\hat{h}}_{i}={\hat{S}}_{i}^{0}\left(\phi ,H\right)\cdot T$$

${\hat{h}}_{i}$ arises from the interactions between a particle and its environment; e.g., solvent, ions, other particles, etc. If ${\hat{S}}_{i}^{0}$ is positive, the presence of particles tends to stabilize the solution locally, and such particles are called *structure makers* In the opposite case, particles are called *structure breakers*. Note that for non-magnetic, neutral particles in the absence of concentration gradient, the particle flux is simplified to:

(108) $${\vec{J}}_{N_{i}}=-\frac{D_{i}\left(\phi_{i}\right)}{k_{B}T}{\hat{S}}_{i}^{0}\left(\phi ,H\right)\vec{\nabla }T$$

The sign of ${\hat{S}}_{i}^{0}$ defines the diffusion direction of the particles under a thermal force (temperature gradient). If ${\hat{S}}_{i}^{0}$ is positive, the particles migrate toward the cold region (*thermophobic*), but if negative, they migrate toward the hot region (*thermophilic*). The notions of thermophilic/thermophobic and of structure maker/breaker are thus equivalent for neutral and non-magnetic particles. This simple relation becomes less straightforward in the case of charged particles [17].

The enthalpies and the Eastman entropies of transfer have been measured experimentally by Ikeda [51,52] in the 1950’s through the Seebeck coefficient measurements, then in a more controlled manner via conductivity for a large number of aqueous ionic species in the 1960s by Snowdon, Turner, Agar and their coauthors [23,49,53,54]. These measurements were made in the framework of Soret effect experiments and the conductivity measurements allowed a more precise determination of the concentration at different points in the measurement cell. However, very little information is available on non-aqueous electrolytes.

#### 3.5.6. Local Field Perturbation Effect on Particle Flux

Even though the applied external field is uniform, the local magnetic field experienced by a single particle can be heterogeneous i.e., field perturbations $\delta \vec{H}$, which can stem from both temperature and concentration variations (in space) within the system. Noting that $\frac{{\vec{H}}_{0}}{\left|{\vec{H}}_{0}\right|}=\vec{h_{0}}$ is constant (uniform applied field), one can re-write up to first order:

(109) $$\left(\frac{\vec{H}}{\left|\vec{H}\right|}\cdot \vec{\nabla }\right)\vec{H}=\vec{h_{0}}\cdot \vec{\nabla }\delta \vec{H}=\vec{\nabla }\left(\vec{h_{0}}\cdot \delta \vec{H}\right)$$

Then $\vec{H}$ can be calculated from Maxwell’s equations.

(110) $$\vec{\nabla }\cdot \vec{B}=0$$

and that

(111) $$\vec{B}=\mu_{0}\left(\vec{H}+\vec{M}\right)$$

the divergence of $\vec{H}$ and $\vec{M}$ must be equal and opposite;

(112) $$\vec{\nabla }\cdot \vec{H}=-\vec{\nabla }\cdot \vec{M}$$

Therefore, one can write the divergence of the magnetic field from the divergence of the corresponding magnetization. $\vec{H}$ and $\vec{M}$ are co-linear and make an angle φ with the unit vector ${\vec{u}}_{z}$ (direction of $\vec{\nabla }T$). According to [39,41,42,44] $\vec{\nabla }\cdot \vec{M}$ can be written as a function of variables ϕ, *T* and $\vec{H}$:

(113)∇→·M→=−∂δHz∂z(114)=∂M∂ϕiT,Hh→0·∇→ϕi+∂M∂Tϕi,Hh→0·∇→T+MH0∇→·δH→+∂M∂Hh→0·∇→H−MHh→0·∇→H

and the divergence of the magnetic field becomes:

(115) $$\frac{\partial \left(\delta H_{z}\right)}{\partial z}=-\cos\left(\varphi \right)\;\frac{{\left(\frac{\partial M}{\partial \phi_{i}}\right)}_{T,H}{\left(\frac{\partial \phi_{i}}{\partial z}\right)}_{x,y}+{\left(\frac{\partial M}{\partial T}\right)}_{\phi_{i},H}{\left(\frac{\partial T}{\partial z}\right)}_{x,y}}{1+{\left(\frac{\partial M}{\partial H}\right)}_{\phi_{i},T}+\left[\frac{M}{H}-{\left(\frac{\partial M}{\partial H}\right)}_{\phi_{i},T}\right]\;\sin^{2}\left(\varphi \right)}$$

The derivative of the magnetization (Equation (62)) with respect to the volume fraction (ϕ) and temperature can be obtained (in the framework of mean field theory) using Maxwell’s relation [40,41]:

(116) $$\begin{array}{cc} {\left(\frac{\partial M}{\partial \phi_{i}}\right)}_{T,H} & =-\frac{1}{\mu_{0}\cdot v_{0}}{\left(\frac{\partial {\tilde{\mu }}_{i}}{\partial H}\right)}_{\phi_{i},T} \end{array}$$

The right-hand side of the equation has already been obtained in Equation (100); i.e.,

(117) $${\left(\frac{\partial M}{\partial \phi_{i}}\right)}_{T,H}=\frac{mL\left(\xi_{e}\right)}{v_{0}\left(1-\lambda \psi_{dd}\phi_{i}L^{\prime }\left(\xi_{e}\right)\right)}$$

The partial derivative of magnetization with respect to temperature is calculated from Equations (62) and (94).

(118)∂M∂Tϕi,H=∂ϕiv0mLξe∂T(119)=−ϕimv0TL′ξeξe1−λψddϕiL′ξe

Lastly, the partial derivative of *M* with respect to magnetic field is obtained from Equation (99) as:

(120) (120)∂M∂Hϕi,T=ϕiv0mL′ξe∂ξe∂H(121)=ϕiψddL′ξe1−λψddϕiL′ξe

This result can be used in the denominator of Equation (115) to calculate:

(122) $$1+{\left(\frac{\partial M}{\partial H}\right)}_{\phi_{i},T}=\frac{1+\left(1-\lambda \right)\phi_{i}\psi_{dd}L^{\prime }\left(\xi_{e}\right)}{1-\lambda \psi_{dd}\phi_{i}L^{\prime }\left(\xi_{e}\right)}$$

Combined together, the final expression of Equation (115) then becomes green (at low field or for φ=0) :

(123) $$\begin{array}{c} \frac{\partial \left(\delta H_{z}\right)}{\partial z}=-\frac{mL\left(\xi_{e}\right)\;\;\cos\left(\varphi \right)}{v_{0}\left(1+\left(1-\lambda \right)\psi_{dd}\phi_{i}L^{\prime }\left(\xi_{e}\right)\right)}{\left(\frac{\partial \phi_{i}}{\partial z}\right)}_{x,y}+\frac{\phi_{i}m}{v_{0}T}\frac{L^{\prime }\left(\xi_{e}\right)\xi_{e}\;\;\cos\left(\varphi \right)}{1+\left(1-\lambda \right)\psi_{dd}\phi_{i}L^{\prime }\left(\xi_{e}\right){\left(\frac{\partial T}{\partial z}\right)}_{x,y}} \end{array}$$

As the concentration gradient is induced by the gradient of temperature, $\vec{\nabla }\phi_{i}$ and $\vec{\nabla }T$ are co-linear, and thus we can define θ the angle between $\vec{\nabla }\phi_{i}$ (or $\vec{\nabla }T$) and $\vec{H}$ (or $\vec{M}$). Then the final expression for the contribution to Equation (83) becomes:

(124) $$\vec{\nabla }\left({\vec{h}}_{0}\cdot \;\delta \vec{H}\right)=-\frac{mL\left(\xi_{e}\right)}{v_{0}\left(1+\left(1-\lambda \right)\psi_{dd}\phi_{i}L^{\prime }\left(\xi_{e}\right)\right)}\;\cos^{2}\left(\varphi \right)\vec{\nabla }\phi_{i}+\frac{\phi_{i}m}{v_{0}T}\frac{L^{\prime }\left(\xi_{e}\right)\xi_{e}}{1+\left(1-\lambda \right)\psi_{dd}\phi_{i}L^{\prime }\left(\xi_{e}\right)}\;\cos^{2}\left(\varphi \right)\vec{\nabla }T$$

This term, clearly due to the concentration and temperature gradients within the system, disappears if $\vec{\nabla }\phi_{i}$ and $\vec{\nabla }T$ are perpendicular to the magnetic field $\vec{H}$. On the other hand, it is maximized when the gradients are parallel to the field.

From Equations (124) in (103), a general expression for the particle flux of $i_{th}$ species can be obtained:

(125) $$\begin{array}{c} {\vec{J}}_{N_{i}}=-D_{i}\left(\phi_{i}\right)[\left(\frac{1}{\chi_{CS}\left(\phi_{i}\right)}-\alpha_{\lambda }\left(\phi_{i},H\right)\right)\vec{\nabla }\frac{\phi_{i}}{v_{0}}+\frac{\phi_{i}}{v_{0}}\frac{{\hat{S}}_{i}^{0}\left(\phi_{i},H\right)+k_{B}S_{1}\left(\phi_{i},H\right)}{k_{B}T}\vec{\nabla }T+\\ \frac{\phi_{i}\mu_{0}m^{2}}{v_{0}^{2}k_{B}T}\frac{L^{2}\left(\xi_{e}\right)}{\left(1-\lambda \psi_{dd}\phi_{i}L^{\prime }\left(\xi_{e}\right)\right)\left(1+\left(1-\lambda \right)\psi_{dd}\phi_{i}L^{\prime }\left(\xi_{e}\right)\right)}\;\cos^{2}\left(\varphi \right)\vec{\nabla }\phi_{i}\\ -\frac{\phi_{i}^{2}}{v_{0}T}\frac{\mu_{0}m^{2}}{v_{0}k_{B}T}\frac{L^{\prime }\left(\xi_{e}\right)L\left(\xi_{e}\right)\xi_{e}}{\left(1-\lambda \psi_{dd}\phi_{i}L^{\prime }\left(\xi_{e}\right)\right)\left(1+\left(1-\lambda \right)\psi_{dd}\phi_{i}L^{\prime }\left(\xi_{e}\right)\right)}\;\cos^{2}\left(\varphi \right)\vec{\nabla }T-\frac{\xi_{i}^{0}e\cdot \phi_{i}}{v_{0}k_{B}T}\vec{E}] \end{array}$$

This can be further simplified to

(126) $$\begin{array}{cc} {\vec{J}}_{N_{i}}= & -D_{i}\left(\phi_{i}\right)[\left(\frac{1}{\chi_{CS}\left(\phi_{i}\right)}-\alpha_{\lambda }\left(\phi_{i},H\right)\right)\vec{\nabla }\frac{\phi_{i}}{v_{0}}+\frac{\phi_{i}}{v_{0}}\frac{{\hat{S}}_{i}^{0}\left(\phi_{i},H\right)+k_{B}S_{1}\left(\phi_{i},H\right)}{k_{B}T}\vec{\nabla }T+\\ & \beta_{\lambda }\left(\phi_{i},H\right)\;\cos^{2}\left(\varphi \right)\vec{\nabla }\frac{\phi_{i}}{v_{0}}-\frac{\phi_{i}}{v_{0}T}S_{2}\left(\phi_{i},H\right)\;\cos^{2}\left(\varphi \right)\vec{\nabla }T-\frac{\xi_{i}^{0}e\cdot \phi_{i}}{v_{0}k_{B}T}\vec{E}] \end{array}$$

with

(127) $$\beta_{\lambda }\left(\phi_{i},H\right)=\frac{\phi_{i}\psi_{dd}L^{2}\left(\xi_{e}\right)}{\left(1-\lambda \psi_{dd}\phi_{i}L^{\prime }\left(\xi_{e}\right)\right)\left(1+\left(1-\lambda \right)\psi_{dd}\phi_{i}L^{\prime }\left(\xi_{e}\right)\right)}$$

and

(128) $$S_{2}\left(\phi_{i},H\right)=\beta_{\lambda }\left(\phi_{i},H\right)\frac{\xi_{e}L^{\prime }\left(\xi_{e}\right)}{L\left(\xi_{e}\right)}$$

**Figure 4:**
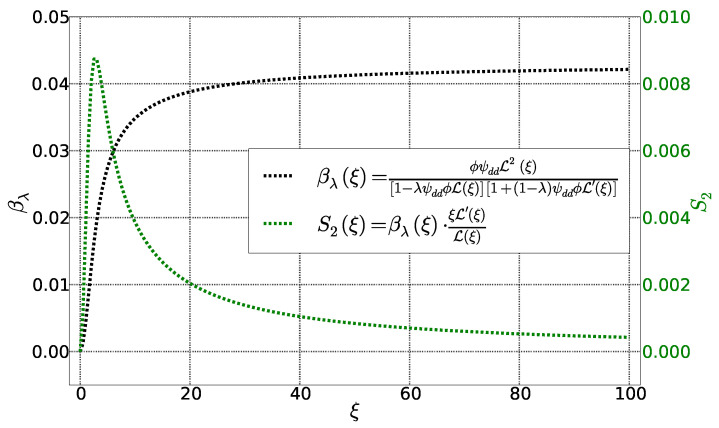
Functions $\beta_{\lambda }$ et $S_{2}$ as a function of Langevin parameter ξ. Other parameters are fixed: ϕ=0,01, λ=0,22 et $\psi_{dd}=4,3$.

These two functions are represented as parametrized Langevin function of ξ in Figure 4. The function $\beta_{\lambda }$ behaves similarly to $\alpha_{\lambda }$. It becomes zero for ξ=0 and saturate when ξ→∞. The function $S_{2}$, on the other hand, approaches zero for ξ=0 and *∞* with an intermediate maximum in between.

We now consider two particular cases that are frequently encountered in magneto-thermodiffusion experiments, i.e., $\vec{\nabla }T⊥\vec{H}$ et $\vec{\nabla }T∥\vec{H}$.

Case I: Field perpendicular to temperature gradient

When the temperature gradient and thus the concentration gradient are perpendicular to the applied magnetic field, the system is homogeneous in the direction parallel to the field. Under such such condition, the particle flux is reduced to:

(129) $${\vec{J}}_{N_{i}}=-D_{i}\left(\phi_{i}\right)\left[\left(\frac{1}{\chi_{CS}\left(\phi_{i}\right)}-\alpha_{\lambda }\left(\phi_{i},H\right)\right)\vec{\nabla }\frac{\phi_{i}}{v_{0}}+\frac{\phi_{i}}{v_{0}}\frac{{\hat{S}}_{i}^{0}\left(\phi_{i},H\right)+k_{B}S_{1}\left(\phi_{i},H\right)}{k_{B}T}\vec{\nabla }T-\frac{\xi_{i}^{0}e\cdot \phi_{i}}{v_{0}k_{B}T}\vec{E}\right]$$

The application of the field still modifies the diffusion of magnetic particles via $\alpha_{\lambda }$ and $S_{1}$, the terms associated to the concentration and the temperature gradients, respectively.

Case II: Field parallel to temperature gradient

In this case, inhomogeneities are present in the direction parallel to the field and Equation (126) becomes, regardless of the field amplitude:

(130) $$\begin{array}{cc} {\vec{J}}_{N_{i}}= & -D_{i}\left(\phi_{i}\right)[\left(\frac{1}{\chi_{CS}\left(\phi_{i}\right)}-\alpha_{\lambda }\left(\phi_{i},H\right)+\beta_{\lambda }\left(\phi_{i},H\right)\right)\vec{\nabla }\frac{\phi_{i}}{v_{0}}+\\ & \frac{\phi_{i}}{v_{0}}\frac{{\hat{S}}_{i}^{0}\left(\phi ,H\right)+k_{B}\left(S_{1}\left(\phi_{i},H\right)-S_{2}\left(\phi_{i},H\right)\right)}{k_{B}T}\vec{\nabla }T-\frac{\xi_{i}^{0}e\cdot \phi_{i}}{v_{0}k_{B}T}\vec{E}] \end{array}$$

#### 3.5.7. Final Expression of Particle Flux in directions parallel and perpendicular to $\vec{H}$

One can introduce a Kronecker-like parameter $\delta_{\vec{\nabla }T\vec{H}}$, in Equation (130):

(131) $$\begin{array}{cc} \delta_{\vec{\nabla }T\vec{H}} & =\left\{\begin{array}{cc} 0 & \mathrm{if}\;\vec{\nabla }T∥\vec{\nabla }\phi_{i}⊥\vec{H}\\ 1 & \mathrm{if}\;\vec{\nabla }T∥\vec{\nabla }\phi_{i}∥\vec{H} \end{array}\right. \end{array}$$

and the following expressions for the diffusion coefficient $D_{i}^{*}$, the Eastman entropy of transfer ${\hat{S}}_{i}$ and the effective charge number $\xi_{i}$:

(132)Di*(ϕi,H)=Di(ϕi)1χCS(ϕi)−αλ(ϕi,H)+δ∇→TH→βλϕi,H(133)S^i(ϕi,H)=S^i0(ϕ,H)+kBS1(ϕi,H)−δ∇→TH→S2(ϕi,H)1χCS(ϕi)−αλ(ϕi,H)+δ∇→TH→βλϕi,H(134)ξi(ϕi,H)=ξi01χCS(ϕi)−αλ(ϕi,H)+δ∇→TH→βλϕi,H

to obtain

(135) $${\vec{J}}_{N_{i}}=-\frac{D_{i}^{*}\left(\phi_{i},H\right)}{v_{0}}\left[\vec{\nabla }\phi_{i}+\phi_{i}\frac{{\hat{S}}_{i}\left(\phi_{i},H\right)}{k_{B}T}\vec{\nabla }T-\frac{\xi_{i}\left(\phi_{i},H\right)e\phi_{i}}{k_{B}T}\vec{E}\right]$$

It needs to be noted; however, that Equation (135) is valid for specific magnetic field direction; parallel or perpendicular with respect to the temperature gradient. Otherwise, Equation (126) (valid only at low field) should be used.

## 4. Calculation of Se and ${\mathrm{Se}}_{\mathrm{int}}$

We are now in the position to calculate the Seebeck coefficient (${\mathrm{Se}}_{\mathrm{int}}$, to be precise) of a thermogalvanic cell containing charged colloidal particles using Equation (135). Exact expressions of ${\mathrm{Se}}_{\mathrm{int}}$ can be found in two distinct states; the initial state, just after the temperature gradient is established across the cell; and at the Soret equilibrium state, (stationary state) when all particle/ion currents come to a halt.

### 4.1. Initial State

Application of a temperature gradient across a homogeneous system exerts a thermal force on all particles, charged or neutral inducing their thermophoretic movements. It is supposed here that the temperature gradient is established instantaneously, i.e., the thermal diffusivity of the suspension is much faster than the diffusion time of ions/particles. The charged particles/ions with the highest thermodiffusion coefficient will diffuse faster than the slower ones, creating an electric field inside the solution ${\vec{E}}_{\mathrm{int}}$. This internal field will “accelerate” the slower particles but “slow down” the faster ones. Therefore, the diffusion coefficients of particles/ions are strongly related to their charges and the global diffusion time of charged species is that of the slowest ones in the solution. The initial electric field when the concentration of all species is still homogeneous can be expressed analytically:

(136) $$\forall i,\;\vec{\nabla }n_{i}=\vec{0}$$

Equation (135) then becomes:

(137) $${\vec{J}}_{N_{i}}=-D_{i}^{*}\left[n_{i}\frac{{\hat{S}}_{i}}{k_{B}T}\vec{\nabla }T-n_{i}\frac{\xi_{i}e}{k_{B}T}{\vec{E}}_{\mathrm{int}}^{ini}\right]$$

Moreover, the total electric current in an open-circuit configuration is null.

(138) $$\sum_{i}z_{i}e{\vec{J}}_{N_{i}}=\vec{0}$$

with $z_{i}$ the charge contribution in the electric charge neutrality of the solution. $z_{i}$ corresponds to the effective static charge of the colloidal particles. Therefore, ${\vec{E}}_{\mathrm{int}}^{ini}$:

(139)∑izie2niDi*ξikBTE→intini=∑izieniDi*S^ikBT∇→T(140)E→intini=∑izieniDi*S^i∑izie2niDi*ξi∇→T(141)E→intini=∑itiS^iξie∇→T

with:

(142) $$t_{i}=\frac{z_{i}\xi_{i}e^{2}n_{i}D_{i}^{*}}{\sum_{i}z_{i}\xi_{i}e^{2}n_{i}D_{i}^{*}}=\frac{\sigma_{i}}{\sigma_{tot}}$$

$t_{i}$ is the transport or Hittorf number, which is the ratio of the conductivity of the ith species to the total conductivity of the solution. The contribution from a given particle/ion is proportional to its Eastman entropy of transfer, that is, the thermal force experienced by the particle/ion. The initial internal Seebeck coefficient is then written as:

(143) $${\mathrm{Se}}_{\mathrm{int}}^{ini}=\sum_{i}t_{i}\frac{{\hat{S}}_{i}}{\xi_{i}e}$$

As we have seen Section 2, there are two components to the Se of a thermogalvanic cell Equation (146). At the initial state, the concentrations of all ions/particles are uniform, including the redox couples. Therefore one can rewrite, up to the first order:

(144) $$\begin{array}{cc} \Delta \mu_{j} & =\frac{\partial \mu_{j}}{\partial n_{j}}\Delta n_{j}+\frac{\partial \mu_{j}}{\partial T}\Delta T=\frac{\partial \mu_{j}}{\partial T}\Delta T=-S_{j}\Delta T \end{array}$$

Additionally, the derivative of the chemical potential with respect to temperature is equal and opposite to the partial entropy. Combined initial Seebeck coefficient is therefore,

(145) $${\mathrm{Se}}^{ini}=\frac{1}{e}\underset{-\Delta_{r}S}{\underset{︸}{\sum_{j}\lambda_{j}S_{j}}}+{\mathrm{Se}}_{\mathrm{int}}$$

where $\Delta_{r}S$ denotes the entropy of the redox half reaction to simplify the expression to:

(146) $${\mathrm{Se}}^{ini}=\frac{1}{e}\left(-\Delta_{r}S+\sum_{i}\frac{t_{i}{\hat{S}}_{i}}{\xi_{i}}\right)$$

The entropy of the redox half reaction $\Delta_{r}S$ can be obtained by the Nernst equation [55], which depends strongly on the ionic strength of the solution [56].

For ordinary ions whose Eastman entropy of transfer is small, the internal Seebeck coefficient is negligibly small compared to the redox reaction entropy. For electrolytes containing charged colloidal particles; however, it can make non-negligible contribution to ${\mathrm{Se}}_{\mathrm{int}}$ as witnessed in certain ferrofluids [16,57]. We note that, the above equation can be used to predict the Seebeck coefficient of electrolytes containing large solute ions and polymers. The latter is of particular interest as the effective charge number of a polymer and its subsequent thermophoretic behavior are known to depend on the pH level of the surrounding solution [58].

### 4.2. Stationary State: Soret Equilibrium

Sufficiently long time after the application of a temperature gradient, the particle flux due to the thermal force is compensated by the opposing flux due to the concentration gradient of all particles and by another flux due to the internal electric field of charged particles/ions. Then the system is said to be in a stationary state, also known as the Soret equilibrium state.

(147) $$\forall i,\;{\vec{J}}_{N_{i}}=\vec{0}$$

The particle flux equation then becomes:

(148) $$\vec{\nabla }n_{i}+n_{i}\frac{{\hat{S}}_{i}}{k_{B}T}\vec{\nabla }T-n_{i}\frac{\xi_{i}e}{k_{B}T}{\vec{E}}_{\mathrm{int}}=\vec{0}$$

for all particles/ions. By exploiting the electrical neutrality at all points inside the cell, the equation above is multiplied by the charge $z_{i}$ and all particle flux equations can be added to give:

(149) $$\sum_{i}z_{i}\vec{\nabla }n_{i}+\sum_{i}z_{i}n_{i}\frac{{\hat{S}}_{i}}{k_{B}T}\vec{\nabla }T-\sum_{i}z_{i}n_{i}\frac{\xi_{i}e}{k_{B}T}{\vec{E}}_{\mathrm{int}}^{Eq}=\vec{0}$$

Note that:

(150)∑izi∇→ni=∑i∇→(zini)(151)=∇→∑izini(152)=0→

The stationary state electric field is then:

(153)∑iziniξiekBTE→intEq=∑iziniS^ikBT∇→T(154)E→intEq=∑iziniS^ie·∑iziξini∇→T

The stationary state electric field is thus independent of the diffusion coefficients of different species and the internal Seebeck coefficient at the Soret equilibrium is:

(155) $${\mathrm{Se}}_{\mathrm{int}}^{Eq}=\frac{\sum_{i}z_{i}n_{i}{\hat{S}}_{i}}{e\cdot \sum_{i}z_{i}\xi_{i}n_{i}}$$

Before calculating the thermogalvanic component of the stationary state Seebeck coefficient, we point out the direct relationship between the ${\mathrm{Se}}_{\mathrm{int}}^{Eq}$ and the Ludwig-Soret coefficient, $S_{T}$:

(156) $$\frac{\vec{\nabla }n}{n}=-S_{T}\vec{\nabla }T$$

$S_{T}$ is the ratio between the concentration gradient of particles/ions and the applied temperature gradient in the stationary state. $S_{T}$ is positive for *thermophobic* particles and negative for *thermophilic* ones. For a given specie *i*, Equation (148) can be rewritten by using the Seebeck coefficient:

(157) $$\vec{\nabla }n_{i}+\left(n_{i}\frac{{\hat{S}}_{i}}{k_{B}T}-n_{i}\frac{\xi_{i}e}{k_{B}T}\cdot {\mathrm{Se}}_{\mathrm{int}}^{Eq}\right)\vec{\nabla }T=\vec{0}$$

and thus:

(158) $$\frac{\vec{\nabla }n_{i}}{n_{i}}=-\left(\frac{{\hat{S}}_{i}}{k_{B}T}-\frac{\xi_{i}e}{k_{B}T}\cdot {\mathrm{Se}}_{\mathrm{int}}^{Eq}\right)\vec{\nabla }T$$

Thus, the Ludwig-Soret coefficient of charged colloidal particle has both thermodiffusive and thermoelectro-diffusive components:

(159) $$S_{T}=\frac{{\hat{S}}_{i}}{k_{B}T}-\frac{\xi_{i}e}{k_{B}T}\cdot {\mathrm{Se}}_{\mathrm{int}}^{Eq}$$

This effect was described in depth by recent work of Würger et co-authors [17,19,30,31,33,59]. Experimentally, it was also demonstrated that for identical and charged nanoparticles, the substitution of counterion (lithium) by another (tetrabutylammonium) can change the same particles from thermophilic to thermophobic [60]. Similar results were also observed for the thermodiffusion of micellar solutions by replacing the dissolved OH${}^{-}$ ions in a solution by Cl${}^{-}$ [61].

Finally, we will now calculate the thermogalvanic contribution to the Seebeck coefficient in the Soret equilibrium state. As all particle currents ceases:

(160) $$\begin{array}{cc} \forall j & \;\vec{\nabla }{\tilde{\mu }}_{j}=\vec{\nabla }\mu_{j}+\vec{\nabla }\mu_{j}^{e}=\vec{\nabla }\mu_{j}-z_{j}e{\vec{E}}_{\mathrm{int}}=\vec{\nabla }\mu_{j}-z_{j}e\cdot {\mathrm{Se}}_{\mathrm{int}}\vec{\nabla }T=-{\bar{\bar{S}}}_{j}\vec{\nabla }T \end{array}$$

By integrating these equations from the hot electrode to the cold and assuming that the transported entropies are constant across the entire cell: For all J equations,

(161) $$\begin{array}{c} \forall j\;\Delta \mu_{j}=z_{j}e\cdot {\mathrm{Se}}_{\mathrm{int}}^{Eq}\Delta T-{\bar{\bar{S}}}_{j}\Delta T \end{array}$$

One can then rewrite the second term of Equation (13) as:

(162)ΔΔrG=∑jνjzj·e·SeintEqΔT−∑jνjS¯¯jΔT(163)=SeintEq−1e∑jνjS¯¯jeΔT

This simplifies the stationary state Seebeck coefficient to

(164) (164)SeEq=SeintEq−SeintEq+1e∑jνjS¯¯j(165)=1e∑jνjS¯¯j

implying that the rearrangement of the redox couple molecules after diffusion screens entirely the internal electric field of the solution. In addition, finally, using the definition of $\bar{\bar{S}}$, one can rewrite the total stationary state Seebeck coefficient as:

(166) $${\mathrm{Se}}^{Eq}=\frac{1}{e}\left(\underset{-\Delta_{r}S}{\underset{︸}{\sum_{j}\nu_{j}S_{j}}}+\sum_{j}\nu_{j}{\hat{S}}_{j}\right)$$

(167) $${\mathrm{Se}}^{Eq}=\frac{1}{e}\left(-\Delta_{r}S+\sum_{j}\nu_{j}{\hat{S}}_{j}\right)$$

At Soret equilibrium, the Seebeck coefficient depends only on the redox reactions with all influence from charged particles/ions lost.

### 4.3. Comparison with Experiments in Ferrofluids

The magneto-thermodiffusion of particles, therefore, impact only the initial state Seebeck coefficient of magnetic nanofluids. Supposing that the ionic strength varies only weakly near the electrodes between the initial and the stationary states, the difference between the stationary and the initial Seebeck coefficients can be expressed simply from Equations (146) and (167); i.e.,

(168) $$\Delta \mathrm{Se}=\frac{1}{e}\left(\sum_{j}\lambda_{j}{\hat{S}}_{j}+\sum_{r}\lambda_{r}{\hat{S}}_{r}-\sum_{i}\frac{t_{i}{\hat{S}}_{i}}{\xi_{i}}\right)$$

Therefore, it is possible to separate the thermogalvanic and the magneto-thermodiffusion components. These models had been applied to describe the experimental data of thermodiffusion and thermoelectric effects in magnetic nanofluids [16,57,62] where ${\hat{S}}_{i}$ of nanoparticles were found to be in the order of 10–100 meV/K per particle, two orders of magnitude larger than typical ions in aqueous electrolytes [54]. Furthermore, the signs of the electrophoretic charge of nanoparticles and the Eastman entropy of transfer with respect to the sign of the thermogalvanic Seebeck coefficient were found to play a decisive role in determining whether the thermodiffusion contribution enhances (in the case of aqueous media with Fe${\left(\mathrm{CN}\right)}_{6}^{3-}$/Fe${\left(\mathrm{CN}\right)}_{6}^{4-}$ redox couple and positively charged nanoparticles) or reduces (in the case of organic solvent media with Ferrocene/Ferrocenium redox couple and negatively charged magnetic nanoparticles). The next test step will be to compare the proposed model to the initial Seebeck coefficient measurement under applied magnetic field. To the best of our knowledge, there are no other experimental reporting of the Seebeck coefficient in ionic nanofluids where relevant physical parameters such as Eastman entropy of transfer and effective charge number of colloidal particles are analyzed.

## 5. Summary

General expressions for particle flux in magnetic nanofluids was derived in the context of magneto-thermoelectric diffusion. The key physical parameters; i.e., diffusion constant, effective charge number and the Eastman entropy transfer are expressed as functions of particle concentration and the applied magnetic field strength. The proposed model can be tested on experimental measurements on the initial Seebeck coefficient in magnetic nanofluids who has been reported to show non-negligible contribution from particle thermodiffusion to the overall production of the thermoelectric potential. It should be noted that in a typical thermogalvanic cell (ΔT of 10–100 kelvin applied across ≈mL of liquid) it can take up to several tens of hours to reach the Soret equilibrium state. Therefore the Seebeck coefficients reported in such thermogalvanic cells correspond to that of (close-to) initial state values. The present model can be relevant for understanding the thermogalvanic effects observed in complex fluids containing charged (magnetic or non-magnetic) particles as well as macro-ions such as ionic liquids. Systematic measurements of the Seebeck coefficient as a function of charged particle concentration and applied magnetic field strength should be used to verify its validity. Furthermore, in comparison to ionic liquids, the thermoelectric property of ionic nanofluids (magnetic or not) is under-explored today. We hope that the present work will serve to motivate the thermogalvanic community to investigate this largely untapped class of complex fluids.
